# Cell-free DNA as a Promising Diagnostic Biomarker in Prostate Cancer: A Systematic Review and Meta-Analysis

**DOI:** 10.1155/2022/1505087

**Published:** 2022-05-27

**Authors:** Cong Zhang, Fan Chao, Shiyu Wang, Dunsheng Han, Gang Chen

**Affiliations:** ^1^Department of Urology, Jinshan Hospital, Fudan University, Shanghai 201508, China; ^2^Department of Surgery, Shanghai Medical College, Fudan University, Shanghai 200032, China

## Abstract

**Objective:**

The detection of cell-free DNA (cfDNA) as a part of “liquid biopsy” of prostate cancer (PCa) has been widely explored. However, its diagnostic value for PCa remains controversial. Based on the data from the latest literature published in the past decade, the present review was conducted to clarify the diagnostic value of cfDNA in PCa.

**Methods:**

The related studies were systematically searched in the databases of PubMed, Embase, Web of Science, and Cochrane Library from January 1, 2010 to December 1, 2020. Sensitivity (SEN), specificity (SPE), and other relative parameters were pooled using a random model.

**Results:**

14 eligible studies with 1049 PCa patients and 973 controls were selected based on the inclusion and exclusion criteria. Results demonstrated that cfDNA showed favorable SPE (0.89, 95% CI: 0.79, 0.94) but unsatisfied SEN (0.56, 95% CI: 0.43, 0.68) in the PCa diagnosis. The positive likelihood ratios (PLR), negative likelihood ratios (NLR), and diagnostic odds ratios (DOR) were 5.1 (95% CI: 3.1, 8.5), 0.49 (95% CI: 0.39, 0.63), and 10 (95% CI: 6, 17), respectively. The summary receiver operating characteristic graph (SROC) with an area under the curve (AUC) of 0.80 (95% CI: 0.76, 0.83) was constructed which indicated favorable diagnostic accuracy for PCa. Results of the subgroup analysis and metaregression analysis reminded “ethnicity” and “methylation” might be sources of heterogeneity. The potential publication bias was not found using Deek's funnel plot asymmetry test (*p* > 0.05).

**Conclusions:**

Our meta-analysis illustrated that the cfDNA could undertake a promising role in the PCa diagnosis.

## 1. Introduction

Prostate cancer (PCa) is the most commonly diagnosed cancer in men, and it alone accounts for more than 20 percentage of newly diagnosed male cancer patients [1]. In 2018, it is reported that 1,276,106 PCa patients were newly diagnosed and 358,989 PCa patients died worldwide [2]. Especially, the morbidity and mortality of PCa have been dramatically increasing in developing countries [3]. Since the late 1980s, the widespread application of prostate-specific antigen (PSA) has been making significant contributions in early diagnosis of PCa; however, due to low specificity, divergences of its effectiveness and the benefit-to-harms ratio have still existed in the medical community [4, 5]. Thus, to remedy the PSA test's defect of low specificity, there is an urgent need for other reliable, accurate, and less invasive methods in screening PCa at an early stage. Circulating cell-free DNA (cfDNA) with its easy accessibility, reliability, and noninvasiveness is regarded as a promising potential biomarker for the early diagnosis of PCa.

CfDNAs are degraded as DNA fragments that are mainly secreted from apoptotic cells or malignant cells [6]. The existence of cfDNA in human body fluid was first officially reported in 1948 by Mandel P et al [7]. The majority of cfDNA in healthy individuals originated from apoptotic cells with a length of 185–200 base pairs [8]. However, in cancer patients, owing to indiscriminate and inadequate digestion of genomic DNA, more uneven and longer DNA fragments are secreted from cancer cells other than the short fragments from apoptosis [9]. Compared to healthy individuals as well as other nonmalignant diseases, the levels of cfDNA are found to be higher in cancer patients [10, 11].

A large number of studies have reported that cancer patients displayed higher levels of cfDNA, such as breast cancer, lung cancer, hepatocellular cancer, colorectal cancer, bladder cancer, and so on [12–16]. Meanwhile, several studies indicated that the abnormal levels of cfDNA showed potential values for the early diagnosis of PCa [17–20]; however, due to the lack of agreement in study methods, sample types, studied genes, and other uncontrollable variations, the consistent conclusions were difficult to be achieved. Therefore, we carried out the present meta-analysis based on the lastest studies published between January 1st, 2010 and December 1st, 2020 with the purpose to elucidate the efficacy of cfDNA in PCa screening and to provide conclusive evidence for the clinical intervention of PCa suspects.

## 2. Materials and Methods

### 2.1. Search Strategy

This study is conducted based on the guidelines of the preferred reporting items for systematic review and meta-analysis (PRISMA) [21]. Based on the database of PubMed, Embase, Web of Science, and Cochrane Library, a comprehensive literature search was performed from January 1, 2010 to December 1, 2020 without any language limitation. The search strategy was completed using a random combination of the following terms: prostate cancer and cell-free DNA. The medical subject heading (MeSH) was employed in the search strategy to avoid the omission of the relevant literature. Furthermore, the meaningful references of eligible articles were also carefully screened in order to obtain additional valuable data.

### 2.2. Inclusion and Exclusion Criteria

The articles complying with all the following criteria were enrolled in the present study: (1) articles evaluated the diagnostic value of cfDNA in serum, plasma, or urine for PCa; (2) the diagnosis of PCa was confirmed by the gold standard test, such as pathology; (3) articles provided sufficient data, for instance, sensitivity (SEN) and specificity (SPE). Studies were excluded whenever the following happened: (1) studies without sufficient publishing data (e.g., SEN or SPE); (2) reviews, case reports, or conference documents and those with invalid data or duplicate publications.

### 2.3. Data Collection and Quality Assessment

Two researchers independently reviewed all eligible studies and carefully extracted data from selected articles. The following disagreements were solved by the discussion of all authors. As for some investigations with insufficient data, the corresponding or the first author were contacted. If no effective response, the study was excluded. The following information was extracted from the original studies: name of the first author, publication year, study location, race, sample size, assay methods, types of cfDNA, sample source, SEN, and SPE.

### 2.4. Risk of Bias Evaluation and Quality Assessment

All selected studies were critically assessed according to the guidelines of Quality Assessment of Diagnostic Accuracy Studies-2 (QUADAS-2) [22]. The four key domains (patient selection, index text, reference standard, and flow and timing) were employed to assess the risk of bias and applicability of the included studies.

### 2.5. Statistical Analysis

The cfDNAs diagnostic sensitivity and specificity, as well as the corresponding true-positive (TP), false-positive (FP), false-negative (FN), and true-negative (TN) were extracted. Positive likelihood ratio (PLR), negative likelihood ratio (NLR), and diagnostic odds ratio (DOR, the odds of PLR to NLR) with the 95% confidence intervals (95% CI) were calculated [23, 24]. Meanwhile, the summary receiver operator characteristic graph (SROC) and the area under the SROC curve (AUC) were plotted based on the SEN and SPE of included articles. Heterogeneity among studies was assessed using the Q-test and *I*^*2*^-statistic. *p* ≤ 0.1 or *I*^*2*^ ≥ 50% represented significant between-study heterogeneity. A random-effects model was employed if heterogeneity existed. Sources of heterogeneity were analyzed using sensitivity and subgroup analysis if necessary. Publication bias was detected by the Deek's test, with *p* < 0.05 indicated statistical significance. Statistical analyses were conducted by RevMan 5.3 (RevMan, the Cochrane Collaboration) and STATA 14.0 software (Stata Corporation, College Station, TX, USA).

## 3. Result

### 3.1. Search Results and Baseline Characteristics

**Figure 1:**
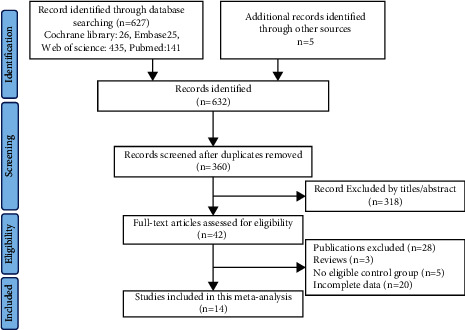
Flow diagram of study selection process.

The stepwise search procedure and results of literature retrieval were illustrated in the flow chart (Figure 1). A total of 632 relative studies were retrieved in the database search and related reference list. After removing duplicates and screening the title and abstract according to the prior selection criteria, 42 articles were adopted to get a full-text review. 3 reviews were excluded, 5 articles were excluded for lacking an eligible control group, and 20 articles were excluded because of insufficient data. Eventually, 14 eligible studies were included in the present meta-analysis with 1049 PCa patients and 973 controls. All articles were published after 2010. Among them, the cfDNA of 4 studies were extracted from plasma [25–28], 8 from serum [17, 18, 29–34], and 2 from urine supernatant [19, 20]. 6 articles assessed the alterations of single-gene methylation [18, 27, 28, 30, 31, 34]. Multiple ethnic groups were included, 6 studies were from Europe [19, 20, 27, 28, 32, 34], 5 were from America [17, 18, 26, 29, 33], and 3 were from Asia [25, 30, 31]. All details of 14 selected studies were presented in Table 1.

### 3.2. Quality Assessment

**Figure 2:**
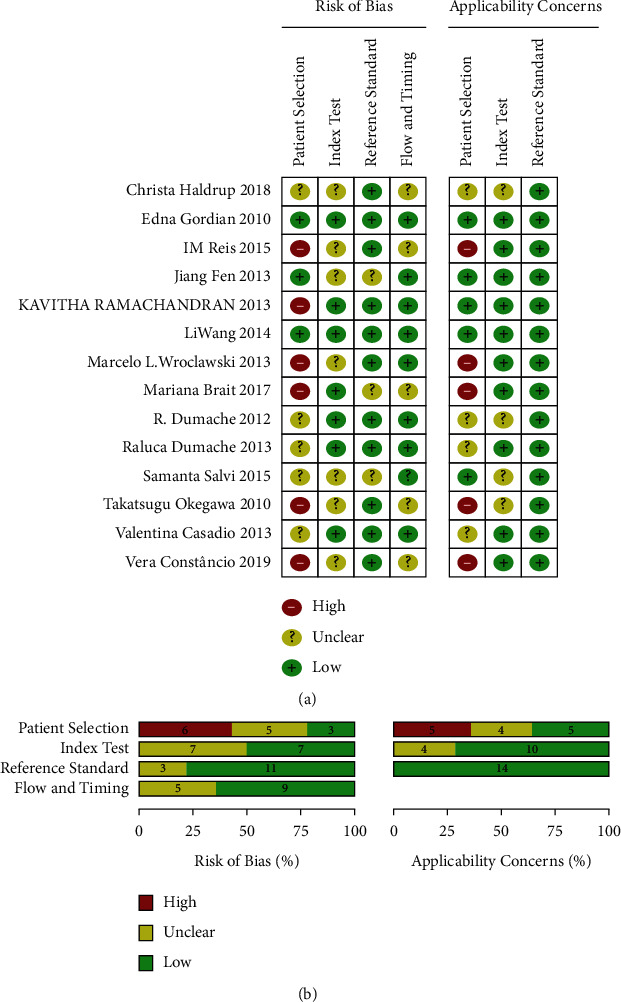
Quality assessments of selected studies based on the QUADAS-2 tool. (a) Risk of bias and application concerns summary; (b) Risk of bias and application concerns graph.

Considering the remarkable heterogeneities of clinical characteristics among studies, the QUADAS-2 was employed to carefully assess the quality of every selected study. After rigorous evaluation, the included studies largely reached the majority of assessment parameters (“Index Text,” “Reference Standard,” and “Flow and Timing”), but the risk of bias and applicability concerns might exist in “Patient Selection.” The results were illustrated in Figure 2.

### 3.3. Diagnostic Accuracy

**Figure 3:**
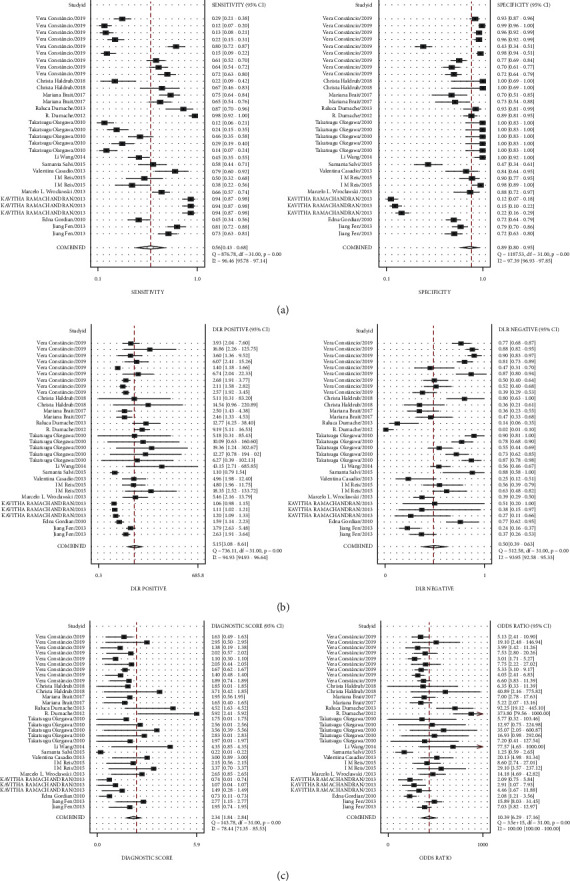
The SEN, SPE, PLR, NLR, and DOR of cfDNA in the PCa diagnosis. (a) The cfDNA's SEN and SPE; (b) the cfDNA's PLR and NLR; (c) the cfDNA's DOR and related diagnostic score.

**Figure 4:**
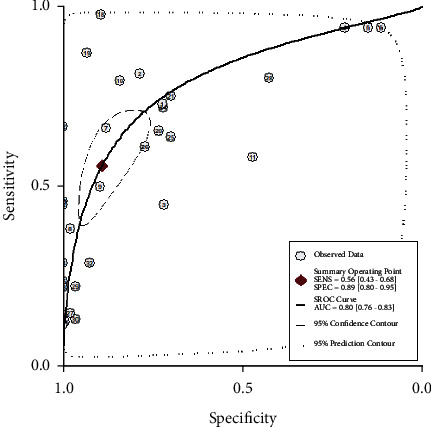
The summary receiver operating characteristic graph of eligible studies.

**Figure 5:**
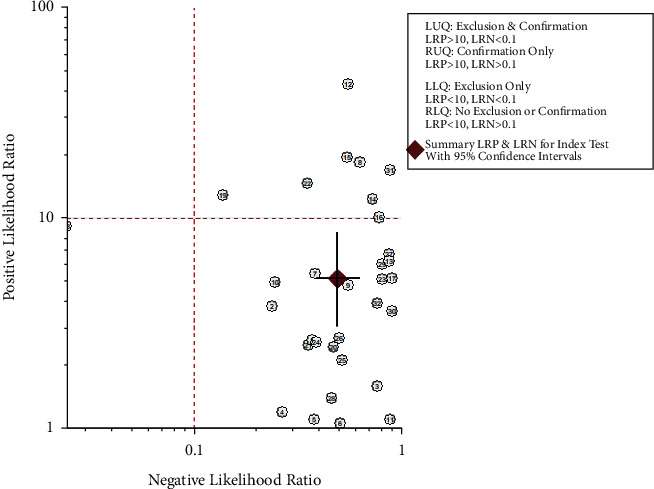
Forest plots of cfDNA's PLR and NLR in the diagnosis of PCa.

**Figure 6:**
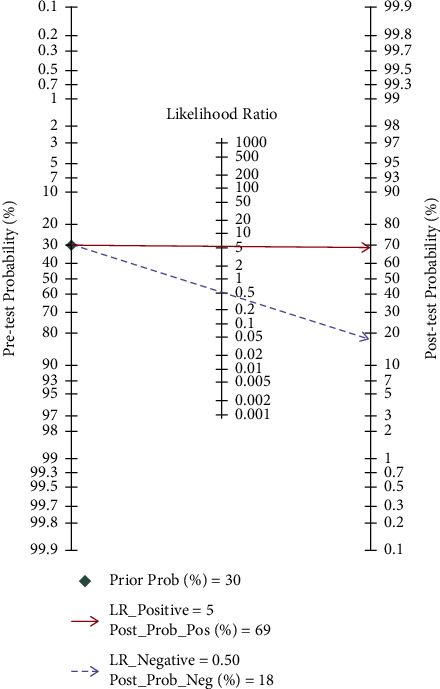
Fagan's nomogram for the post-test probabilities calculation.

To elucidate the diagnostic value of cfDNA for PCa, the pooled SEN and SPE were explored using forest plots. The results indicated that pooled SEN and SPE were 0.56 (95% CI = 0.43–0.68) and 0.89 (95% CI = 0.79–0.94), respectively. At the same time, the pooled DOR (10.34, 95% CI: 6.27, 17.06), PLR (5.09, 95% CI: 3.06, 8.48), and NLR (0.49, 95% CI: 0.39, 0.63) were presented. However, significant heterogeneities among included studies were discovered (Figure 3). Therefore, we further conducted the random-effects model to calculate the pooled estimates. Based on the selected studies, the summary receiver operating characteristic graph (SROC) was adopted to assess the significance of cfDNA in distinguishing the PCa from control. Results showed that the summary SEN and SPE were 0.56 (95% CI: 0.43, 0.68) and 0.89 (95% CI = 0.79, 0.94), respectively, which were consistent with the data of forest plots. The area under the curve (AUC) was 0.80 (95% CI: 0.76, 0.83), demonstrating satisfactory diagnostic performance (Figure 4). The overall PLR, NLR, and DOR were 5.1 (95% CI: 3.1, 8.5), 0.49 (95% CI: 0.39, 0.63), and 10 (95% CI: 6, 17), respectively (Table 2), and the forest plot of PLR and NLR were created at the same time (Figure 5). A Fagan's Nomogram was constructed as well in order to predict the index test's clinical utility (Figure 6).

### 3.4. Subgroup Analysis

Based on the ethnicity, methylation, and sample source, the subgroup analyses were made and the results were illustrated in Table 2. According to the subgroup analysis of ethnicity, we found that Americans had the highest sensitivity (SEN = 0.75%), while the Asian owned the excellent specificity (SPE = 100%). However, it is interesting that Europeans possessed the largest AUC compared to the other two (AUC = 0.83). In the subgroup of the sample types, the highest sensitivity was obtained from the urine supernatant sample (SEN = 0.65) and the best specificity (SPE = 0.95) was found in the serum sample. Unfortunately, due to the small sample size of urine, only consisting of two studies, the AUC of this subgroup was unaccessible. Compared to nonmethylated genes, the methylations performed better SPE (0.95) and more advantageous AUC (0.84), but poorer SEN (0.44).

### 3.5. Heterogeneity and Metaregression Analysis

**Figure 7:**
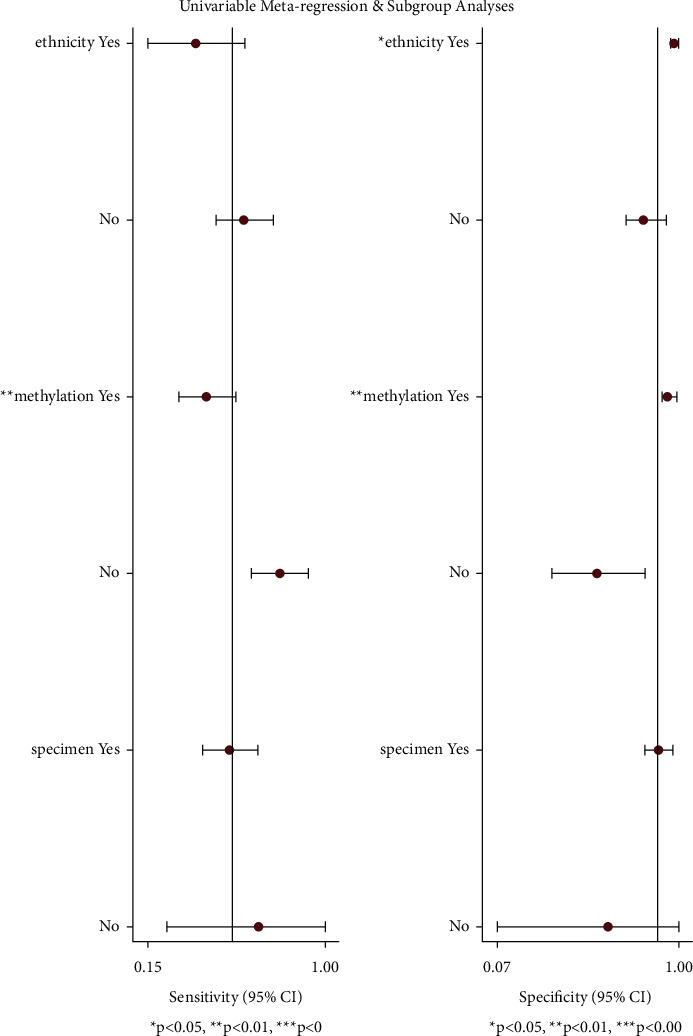
Forest plot of univariable metaregression based on sensitivity and specificity.

In order to further explore the potential source of between-studies heterogeneity, the metaregression analysis was constructed. The following items were included in the analysis: ethnicity, methylation, and specimen. Results of mate-regression analysis suggested that “methylation” might lead heterogeneities to the both “sensitivity” and “specificity,” while “ethnicity” merely accounts for the latter (Figure 7).

### 3.6. Sensitivity Analysis and Publication Bias

**Figure 8:**
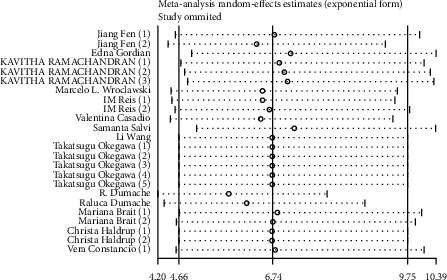
Sensitivity analysis of selected studies.

**Figure 9:**
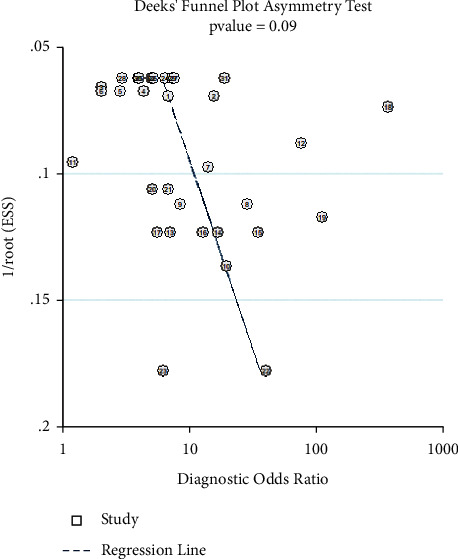
Deek's test for the assessment for potential publication bias of selected studies.

Then the influence of individual trials on the pooled results was assessed by sensitivity analysis, and no significant differences were found after omitting any of the selected studies (Figure 8). The publication bias of included studies was assessed using the Deek's test, and no obvious bias was observed (Figure 9). Those results declared that our conclusions were convinced.

## 4. Discussion

With the aging of the global population, PCa is a growing threat to male health worldwide, and its incidence is estimated to be doubled by 2030 [35]. PSA as a classical screening biomarker is widely used in the PCa screening in the past decades [36]. Although the widespread clinical applications of the PSA have created a remarkable contribution to the early detection of PCa, the several issues related to it in clinical practice have to be concerned. First, the level of PSA is susceptible to several interference factors, such as benign prostatic hyperplasia (BPH) and prostatitis, which can both increase the PSA concentration and lead to unnecessary intervention in noncancer patients. Another problem is that an increasing number of clinically significant PCa patients fail to be filtrated using PSA only [37]. Thus, novel molecular markers that can effectively remedy deficiencies of the present diagnosis ways are still urgently needed. CfDNA originating from multiple tissues has enormous clinical potential [38], and this liquid biopsy-based detection tool offers substantial advantages in early detection and dynamic monitoring of diseases [39]. Thus, first combining blood and urine samples, we performed the present detailed systematic review to evaluate the effect of cfDNA in PCa diagnosis.

Based on the inclusion and exclusion criteria, a total of final 14 studies were included in the present meta-analysis. The risk of bias evaluation and quality assessment of all included studies were critically assessed according to the guidelines of Quality Assessment of Diagnostic Accuracy Studies-2 (QUADAS-2). Among those assessment parameters, the “Index Text,” “Reference Standard,” and “Flow and Timing” were without risk of bias and applicability concerns. However, the risk of bias and applicability concerns might exist in “Patient Selection,” and this result might be caused by the following. The included studies were performed in different institutions of many countries and involved racial differences, which inevitably led to the risk of bias and applicability concerns in “Patient Selection.”

The subgroup analyses of the diagnostic value of cfDNA for PCa were made based on the ethnicity, sample types, and methylation whether or not. In ethnicity analyses, we found the diagnostic sensitivity of cfDNA in Americans was best (SEN = 0.75) but specificity was relatively poor (SPE = 0.63), while the cfDNA in Asians showed the best diagnostic specificity (SPE = 1.00) but the worst diagnostic sensitivity (SEN = 0.38). However, it was interesting that cfDNA in Europe had the best diagnostic value for PCa (AUC = 0.83). The difference between SEN and SPE in terms of ethnicity could be ascribed to a difference in study methodology such as cfDNA detection methods. Furthermore, the sample sizes in the subgroups of ethnicity were small (5 studies focusing on Americans, 3 studies focusing on Asians, and 6 studies focusing on Europeans), leading to imprecise diagnostic sensitivity and specificity, and this might be another reason that contributed to this difference. In the analyses of sample types, cfDNA in urine exhibited the best sensitivity (SEN = 0.65), but it was noteworthy that the reliability of this result might be poor due to the too small sample size. Furthermore, in the subgroup of plasma and serum, the cfDNA exhibited moderate diagnostic sensitivity (SEN = 0.55), specificity (SPE = 0.90), and values (AUC = 0.81) for PCa compared to the plasma or serum subgroup. Compared to the nonmethylation group, the cfDNA in the methylation group showed higher specificity (SPE = 0.95) and better diagnostic value for PCa (AUC = 0.84). It was noteworthy that some genes in methylation status showed favorable diagnostic value for PCa. The methylation status of GSTP1 demonstrated a favorable diagnostic sensitivity (SEN = 0.87) and specificity (SPE = 0.93) [28]. The prognostic sensitivity and specificity of RAR*β*2 methylation for PCa were 0.98 and 0.89, respectively [34]. However, the common limitation of those studies was a small sample size [28].

The present study revealed the pooled diagnostic sensitivity and specificity of cfDNA for PCa were 0.56 and 0.89, respectively. Although the sensitivity of cfDNA was less than ideal, the specificity was higher. Considering the high sensitivity and low specificity of PSA [40], the combination of cfDNA and PSA could efficiently detect PCa in an early stage. It is of interest that the pooled sensitivity of the whole study was lower than the sensitivity of one study. We thought it was the “bias in Patient Selection” that caused the differences. The high sensitivity of some studies may be caused by the “bias in patient selection.” A single center study had included a relatively small size and involved a single ethnicity, which might have led to the “bias in patient selection” and further acquired a relatively higher sensitivity accompanying with applicability concerns. The present meta-analysis was performed to demonstrate the diagnostic value of cfDNA for PCa based on multiple studies including a large sample size; therefore, the pooled SEN might be different from or even worse than the SEN of one single study. At the same time, the PLR (5.09), NLR (0.49), and DOR (10.34) were explored as well. With a DOR value of 10.34, it indicated that cfDNA owned outstanding discriminatory test performance and could efficiently differentiate PCa from control cases. Based on Fagan's nomogram, supposing the pretest probability was 30 percent, the post-test probability of cfDNA's positive diagnostic results for PCa was 69 percent, and the positive likelihood ratio was 3. The probability of post-test negative results was reduced to 18 percent with a negative likelihood ratio of 0.50. Above all results strongly proved the stable value of cfDNA in the diagnosis of PCa. The overall diagnostic performance of cfDNA was further assessed by employing the SROC curve. As shown in Figure 4, the AUC was 0.80 which exhibited satisfactory diagnostic effect. However, the significant heterogeneity between-studies existed, but the typical “should arm” was not found in the ROC plane indicating nonthreshold effects in the heterogeneity of the present study. In order to explore the sources of heterogeneity, the univariable metaregression and subgroup analyses were further carried out according to ethnicity, sample types, and methylation. Taking account of the results of the two analyses, we predicted that the sources of heterogeneity might result from differences in methylation and ethnicity, mainly from the former.

The results of the present analysis illustrated the meaningful diagnostic value of cfDNA for PCa, but several limitations that existed in the present study should be noticed. First, almost all of the included studies were retrospective and did not explicitly declare compliance with standard blind methods in the study design and implementation which might generate potential bias in “patient selection.” Second, for the majority of the included studies, the cutoff values of cfDNA for the PCa diagnosis were different from others. The phenomenon was not difficult to understand due to various genes, different analytical methods, and the subjectiveness of the operatives. Third, the enrolled studies' sample sizes in the present meta-analysis were not large enough, especially studies based on the urine sample, which might interfere with the diagnostic accuracy of cfDNA and restrict its reproducibility. Given all the above concerns, the standardized clinical studies with a larger sample size are expected to be established in the future.

## 5. Conclusions

As the first meta-analysis based on the sample from blood and urine, the results of present study indicated that cfDNA owned a promising role to be an ideal biomarker for the early PCa diagnosis, especially as the adjuvant tool of the PSA screening due to its outstanding specificity. However, it should be emphasized there remained several limitations described above. Therefore, high-quality studies with large enough samples are needed to be conducted in the future.

## Figures and Tables

**Table 1:** Characteristics of the included studies.

Country	Ethnicity	Case (Age, N)	Control (Age, N)	Control type	Target	Methylation	Specimen	Assay methods	Cutoff	Sensitivity	Specificity
China	Asian	N/A, 96	N/A, 112	Benign disease	CfDNA	No	Plasma	qPCR (quantitative)	0.864	73.20%	72.70%
China	Asian	N/A, 96	N/A, 112	Benign disease	CfDNA	No	Plasma	qPCR (quantitative)	0.91	81.70%	78.80%
American	American	65.5, 89	62.9, 163	Benign disease	GSTP1	No	Serum	qPCR (quantitative)	180 ng/ml	44.90%	71.80%
American	American	65.5, 85	63.1, 156	Benign disease	CfDNA	No	Serum	qPCR (quantitative)	180 ng/ml	94.10%	21.80%
American	American	65.5, 85	63.1, 156	Benign disease	CfDNA	No	Serum	Pico green	53.1 ng/ml	94.10%	15.40%
American	American	65.5, 85	63.1, 156	Benign disease	CfDNA	No	Serum	Uv absorbance	N/A	94.10%	11.50%
Brazil	American	66.81, 133	66.40, 33	Benign disease	CfDNA	No	Plasma	Pico green dsDNA quantification reagent kit	140 ng/ml	66.20%	87.90%
American	American	64.9, 34	64.5, 48	Benign disease	GADD45a	Yes	Serum	Ms-SNuPE	89.6	38.20%	97.90%
American	American	64.9, 34	64.5, 48	Benign disease	CfDNA	No	Serum	qPCR (quantitative)	188	50.00%	89.58%
Italy	European	N/A, 29	N/A, 25	Benign disease	CfDNA	No	Urine Supernatant	qPCR (quantitative)	0.04 ng/ul	79.00%	84.00%
Italy	European	N/A, 55	N/A, 55	Benign disease	CfDNA	No	Urine Supernatant	qPCR (quantitative)	0.04 ng/ul	58.00%	47.00%
China	Asian	N/A, 98	N/A, 47	Benign disease	CDH13promoter	Yes	Serum	Methylation-specific polymerase chain reaction (MSP)	N/A	44.90%	100.00%
Japan	Asian	72.2, 76	69.4, 20	Benign disease	Hypermethylation APC	Yes	Serum	Methylation-specific polymerase chain reaction (MSP)	0.002	15.00%	100.00%
Japan	Asian	72.2, 76	69.4, 20	Benign disease	Hypermethylation GSTP1	Yes	Serum	Methylation-specific polymerase chain reaction (MSP)	0.147	29.00%	100.00%
Japan	Asian	72.2, 76	69.4, 20	Benign disease	Hypermethylation MDR1	Yes	Serum	Methylation-specific polymerase chain reaction (MSP)	0.037	46.00%	100.00%
Japan	Asian	72.2, 76	69.4, 20	Benign disease	Hypermethylation RASSF1	Yes	Serum	Methylation-specific polymerase chain reaction (MSP)	0.119	24.00%	100.00%
Japan	Asian	72.2, 76	69.4, 20	Benign disease	Hypermethylation PTGS2	Yes	Serum	Methylation-specific polymerase chain reaction (MSP)	0.091	12.00%	100.00%
Romania	European	68.0, 91	64.0, 94	Benign disease	RAR*β*2	Yes	Serum	Methylation-specific polymerase chain reaction (MSP)	N/A	98.00%	89.00%
Romania	European	64.0, 31	68.0, 44	Benign disease	GSTP1	Yes	plasma	methylation-specific polymerase chain reaction (MSP)	N/A	87.10%	93.18%
American	American	59.0, 84	N/A, 30	AC	MCAM	Yes	Serum	Pyrosequencing	N/A	66.00%	73.00%
American	American	59.0, 84	N/A, 30	AC	MCAM/*ERα*/Er*β*	Yes	Serum	Pyrosequencing	N/A	75.00%	70.00%
Denmark	European	N/A, 27	N/A, 10	BPH	CCDC181/ST6GALNAC3/HAPLN3	Yes	Serum	qMSP	N/A	67.00%	100.00%
Denmark	European	N/A, 27	N/A, 10	BPH	ZNF660	Yes	Serum	ddMSP	N/A	22.00%	100.00%
Portugal	European	71.0, 121	57.0, 136	AC	FOXA1/RAR*β*2/RASSF1A/GSTP1	Yes	Plasma	Multiplex qMSP	N/A	72.00%	72.00%
Portugal	European	71.0, 121	57.0, 136	AC	FOXA1/RAR*β*2/RASSF1A)	Yes	Plasma	Multiplex qMSP	N/A	64.00%	70.00%
Portugal	European	71.0, 121	57.0, 136	AC	FOXA1	Yes	Plasma	Multiplex qMSP	N/A	61.00%	77.00%
Portugal	European	71.0, 121	57.0, 136	AC	GSTP1	Yes	Plasma	Multiplex qMSP	N/A	15.00%	98.00%
Portugal	European	71.0, 121	57.0, 136	AC	HOXD3	Yes	Plasma	Multiple qMSP	N/A	80.00%	43.00%
Portugal	European	71.0, 121	57.0, 136	AC	RAR*β*2	Yes	Plasma	Multiplex qMSP	N/A	22.00%	96.00%
Portugal	European	71.0, 121	57.0, 136	AC	RASSF1A	Yes	Plasma	Multiplex qMSP	N/A	13.00%	96.00%
Portugal	European	71.0, 121	57.0, 136	AC	SEP-19	Yes	Plasma	Multiplex qMSP	N/A	12.00%	99.00%
Portugal	European	71.0, 121	57.0, 136	AC	SOX17	Yes	Plasma	Multiplex qMSP	N/A	29.00%	93.00%

BPH, benign prostatic hyperplasia; AC, asymptomatic control; cfDNA, cell-free DNA; qMSP, quantitative methylation-specific PCR; ddMSP, methylation-specific droplet digital PCR; N/A, not available; N, number.

**Table 2:** Summary diagnostic performance of cell-free DNA for prostate cancer.

Group	Subgroup	SEN (95% CI)	SPE (95% CI)	PLR (95% CI)	NLR (95% CI)	DOR (95% CI)	AUC (95% CI)
Ethnicity	Asian	0.38 (0.22, 0.58)	1.00 (0.77, 1.00)	176.9 (1.6, 19814.7)	0.62 (0.45, 0.84)	287 (3, 29034)	0.81 (0.77, 0.84)
	European	0.53 (0.34, 0.71)	0.90 (0.80, 0.95)	5.3 (2.9, 9.6)	0.53 (0.36, 0.76)	10 (5, 21)	0.83 (0.79, 0.86)
	American	0.75 (0.56, 0.87)	0.63 (0.35, 0.84)	2.0 (1.2, 3.5)	0.40 (0.29, 0.35)	5 (3, 9)	0.76 (0.72, 0.79)

Sample types	Plasma and serum	0.55 (0.41, 0.68)	0.90 (0.81, 0.95)	5.5 (3.2, 9.4)	0.50 (0.39, 0.64)	11 (7, 18)	0.81 (0.77, 0.84)
	Plasma	0.50 (0.33, 0.67)	0.88 (0.78, 0.94)	4.3 (2.8, 6.4)	0.57 (0.43, 0.75)	8 (5, 11)	0.79 (0.75, 0.82)
	Serum	0.58 (0.39, 0.76)	0.95 (0.77, 0.99)	11.6 (2.8, 48.7)	0.44 (0.29, 0.66)	26 (7, 100)	0.84 (0.80, 0.87)
	Urine supernatant	0.65 (−)	0.59 (−)	1.59 (−)	0.59 (−)	2.69 (−)	—

Methylation	Yes	0.44 (0.31, 0.58)	0.95 (0.89, 0.98)	8.7 (4.5, 16.6)	0.59 (0.47, 0.75)	15 (7, 29)	0.84 (0.80, 0.87)
	No	0.78 (0.64, 0.88)	0.59 (0.36, 0.78)	1.9 (1.2, 2.9)	0.37 (0.26, 0.53)	5 (3, 9)	0.76 (0.73, 0.80)

Overall		0.56 (0.43, 0.68)	0.89 (0.80, 0.95)	5.1 (3.1, 8.6)	0.49 (0.39, 0.63)	10 (6, 17)	0.80 (0.76, 0.83)

SEN, sensitivity; SPE, specificity; PLR, positive likelihood ratios; PLR, positive likelihood ratios; NLR, negative likelihood ratios; DOR, diagnostic odds ratios; AUC, the area under curve.

## Data Availability

The data used to support the findings of this study are included within the article.

## Abbreviation

PCa: Prostate cancer
cfDNA: Cell-free DNA
SEN: Sensitivity
SPE: Specificity
PLR: Positive likelihood ratios
NLR: Negative likelihood ratios
DOR: Diagnostic odds ratios
AUC: The area under the curve.
